# Blood pressure variability, heart functionality, and left ventricular tissue alterations in a protocol of severe hemorrhagic shock and resuscitation

**DOI:** 10.1152/japplphysiol.00348.2018

**Published:** 2018-07-12

**Authors:** Marta Carrara, Giovanni Babini, Giuseppe Baselli, Giuseppe Ristagno, Roberta Pastorelli, Laura Brunelli, Manuela Ferrario

**Affiliations:** ^1^Department of Electronics, Information, and Bioengineering, Politecnico di Milano, Milan, Italy; ^2^Istituto di Ricerche Farmacologiche Mario Negri IRCCS, Milan, Italy; ^3^Department of Pathophysiology and Transplantation, University of Milan, Milan, Italy

**Keywords:** cardiovascular autonomic control, hemorrhagic shock, metabolomics, myocardial sufferance, resuscitation

## Abstract

Autonomic control of blood pressure (BP) and heart rate (HR) is crucial during bleeding and hemorrhagic shock (HS) to compensate for hypotension and hypoxia. Previous works have observed that at the point of hemodynamic decompensation a marked suppression of BP and HR variability occurs, leading to irreversible shock. We hypothesized that recovery of the autonomic control may be decisive for effective resuscitation, along with restoration of mean BP. We computed cardiovascular indexes of baroreflex sensitivity and BP and HR variability by analyzing hemodynamic recordings collected from five pigs during a protocol of severe hemorrhage and resuscitation; three pigs were sham-treated controls. Moreover, we assessed the effects of severe hemorrhage on heart functionality by integrating the hemodynamic findings with measures of plasma high-sensitivity cardiac troponin T and metabolite concentrations in left ventricular (LV) tissue. Resuscitation was performed with fluids and norepinephrine and then by reinfusion of shed blood. After first resuscitation, mean BP reached the target value, but cardiovascular indexes were not fully restored, hinting at a partial recovery of the autonomic mechanisms. Moreover, cardiac troponins were still elevated, suggesting a persistent myocardial sufferance. After blood reinfusion all the indexes returned to baseline. In the harvested heart, LV metabolic profile confirmed the acute stress condition sensed by the cardiomyocytes. Variability indexes and baroreflex trends can be valuable tools to evaluate the severity of HS, and they may represent a more useful end point for resuscitation in combination with standard measures such as mean values and biological measures.

**NEW & NOTEWORTHY** Autonomic control of blood pressure was highly impaired during hemorrhagic shock, and it was not completely recovered after resuscitation despite global restoration of mean pressures. Moreover, a persistent myocardial sufferance emerged from measured cardiac troponin T and metabolite concentrations of left ventricular tissue. We highlight the importance of combining global mean values and biological markers with measures of variability and autonomic control for a better characterization of the effectiveness of the resuscitation strategy.

## INTRODUCTION

Hemorrhage and unresolved hemorrhagic shock (HS) still represent the leading cause of mortality after trauma in both civilian and military settings, with the majority of deaths occurring because of the inability to control bleeding and to effectively resuscitate hemorrhage patients (27, 67).

Therefore, much effort has been spent on investigation of the fundamental underlying pathophysiology of HS in order to develop innovative approaches or to discover new biological parameters capable of detecting the severity of blood loss, controlling bleeding, and guiding resuscitation.

HS is a form of hypovolemic shock in which an acute reduction in central blood volume causes organ hypoperfusion and consequently an inadequate oxygen supply at the cellular level. Clinical signs of this condition are severe hypotension and hypoxia, pronounced tachycardia and tachypnea, diffused coagulopathy, hypothermia, and metabolic acidosis (14).

During progressive hemorrhage, physiological compensatory mechanisms are usually elicited in trying to maintain homeostasis. Indeed, the cardiovascular system activates physiological responses with the aim of maintaining cerebral oxygenation and blood supply to central organs; for example, neuroendocrine-mediated modifications of peripheral vascular resistance cause a redistribution of fluids that leads to nonuniform regional blood loss. Other compensatory mechanisms consist of an increase in heart rate (HR) and myocardial contractility to increase cardiac output (CO) (62).

The role of the sympathetic autonomic nervous system (ANS) has been demonstrated to be crucial during bleeding to prevent collapse through reflex tachycardia and peripheral vasoconstriction (6, 17, 18, 23, 24, 60). Schiller et al. (62) described a dynamic coupling between arterial blood pressure (ABP) and sympathetic outflow oscillations during progressive central hypovolemia. A decrease in ABP quickly initiates an increase in traffic of sympathetic nerve impulses by decreasing inhibitory afferent activity to the nucleus of the solitary tract; the subsequent arterial vasoconstriction results in increased vascular tone and compensatory elevation in ABP, activating a baroreflex-mediated feedback reduction in sympathetic outflow. This baroreflex-mediated phenomenon of oscillatory coupling between blood pressure (BP) and sympathetic activity represents an important compensatory mechanism during hypovolemia, and there is evidence that it may be lost at the point of hemodynamic decompensation (60). These dynamics underscore the physiological importance of measuring oscillations of BP rather than relying on an average trend. Analysis of BP variability could help in understanding the extent of ANS activation in response to volume depletion, the severity of hemorrhage, whether the patient is approaching shock, and, at the same time, whether he is likely to recover, i.e., when the resuscitation is effective.

Standards of resuscitation for prehospital hemorrhagic trauma patients include administration of fluids to stabilize BP and vascular volume before blood transfusion and surgical repair. However, after the replacement of blood loss the consequent restoration of CO and ABP does not accurately reflect the effectiveness of treatment (52, 58). In fact, overzealous resuscitation with crystalloids dilutes oxygen-carrying capacity and clotting factor concentrations, thus exacerbating coagulopathy, inflammation, and hypoxia (14, 38).

For this reason, resuscitation science has tried to identify other surrogates able to assess cardiocirculatory status and tissue perfusion so as to better depict shock severity and response to treatment (e.g., lactate, mixed venous saturation, base deficit, and oxygen debt) (3, 59).

In this study, we analyzed the cardiovascular signals recorded during a protocol of severe hemorrhage and resuscitation with the aim of characterizing the compensatory response to hypovolemia and fluid repletion in terms of autonomic-mediated changes in BP variability and HR variability (HRV).

Furthermore, hemorrhage is known to induce a sort of myocardial injury (15), and the reduction in heart size with central hypovolemia stimulates the release of other vasoactive and volume regulatory hormones, such as arginine vasopressin and renin. Vasoactive hormonal responses to hemorrhage in animals may also be species dependent; for example, Thrasher (69) confirmed that arterial baroreflexes control vasopressin but not renin release during graded hypotension in the dog.

For these reasons, we assessed the effects of severe hemorrhage on heart functionality and cardiac tissue by integrating the hemodynamic findings with measurements of plasma high-sensitivity cardiac troponin T (hs-cTnT) and metabolite concentrations in left ventricular (LV) tissue.

## MATERIALS AND METHODS

### Study Design and Experimental Procedure

#### Animal preparation.

Nine male pigs (36 ± 2 kg) received anesthesia by intramuscular injection of ketamine (20 mg/kg), completed by ear vein injection of propofol (2 mg/kg) and sufentanil (0.3 μg/kg) and then maintained over the whole experiment with continuous intravenous administration of propofol (1–3 mg·kg^−1^·h^−1^), midazolam (2–4 mg·kg^−1^·h^−1^), and sufentanil (0.3 μg·kg^−1^·h^−1^). Animals were mechanically ventilated with a tidal volume of 10 ml/kg^−1^ and inspired O_2_ fraction of 0.21 during baseline and 0.3 during and after the shock phase; respiratory frequency was adjusted to maintain the end-tidal partial pressure of CO_2_ (Pco_2_) values in the range 35–40 mmHg. A 7-Fr catheter was placed in the descending aorta from the left femoral artery for blood sample collection. Another Millar Mikro-Tip pressure catheter was inserted in the right femoral artery for continuous monitoring of ABP. Continuous acquisition of central venous pressure in the right atrium, pulmonary arterial pressure, and CO through thermodilution technique was obtained by means of an endovascular pentalumen 7-Fr catheter placed in the pulmonary artery from the right femoral vein. Continuous monitoring of LV pressure and volume was achieved by means of a Millar Mikro-Tip pressure catheter inserted in the LV cavity from the right carotid artery. Finally, a 14-Fr catheter was placed in the abdominal artery from the left femoral artery for blood withdrawal during the induction of shock, and a 14-Fr catheter was inserted in the left femoral vein for the successive blood reinfusion. The study was reviewed and approved by the Institutional Review Board and the governmental institution (Italian Ministry of Health).

#### Acute hemorrhagic shock model.

After baseline measurements, animals were randomized to one of two study groups: *1*) HS and resuscitation (*n* = 6) and *2*) sham-treated control (*n* = 3). Bleeding was induced by withdrawal of blood from the left femoral artery with a peristaltic pump at a rate of 20 ml/min over an interval of 60 min, until mean arterial pressure (MAP) reached values of 40 ± 5 mmHg. The hypoxic state with the consequent metabolic alteration was confirmed by serial blood lactate measurements. After 2 h of the shock condition, animals were resuscitated with a two-step procedure. Initially, fluid (normal saline) and vasopressor (norepinephrine) were administered to restore MAP of at least 60 mmHg and a pulse pressure variation < 12%. Then, shed blood was reinfused with the aid of a peristaltic pump over an interval of 30 min. After 1 h of observation, animals were euthanized by intravenous injection of Tanax (1 ml/10 kg) and heart biopsies from the LV were taken for histological, biochemical, and omics analyses.

For sham-treated animals the preparation was identical, but they did not undergo bleeding, fluid resuscitation, or blood reinfusion. One of six HS animals was excluded because of elevated blood pressure at baseline (MAP of 130 mmHg).

Animals were studied at four relevant time points: at baseline (T1), after the development of HS (T2), after fluid and vasopressor resuscitation (T3), and after blood reinfusion (T4).

At each time point a bolus of phenylephrine (3 μg/kg) and a bolus of epinephrine (10 μg) were intravenously administered to elicit the response of the ANS. Arterial and venous blood samples were collected for blood gas analysis and laboratory analyses (i-STAT System; Abbott Laboratories, Princeton, NJ). Plasma hs-cTnT was measured in a central laboratory by electrochemiluminescence immunoassay using commercial reagents (Elecsys 2010; Roche Diagnostics).

After death, a thoracotomy was immediately performed, the heart was exposed, and the LV anterior free wall opposite to septum 1 cm below mitral valve level was removed and collected. Samples of 50–60 mg comprising the entire thickness of the muscle (endocardium to epicardium) were rinsed with protease inhibitor solution and stored at −80°C.

### Clinical Data

Clinical variables collected at each time point were the following: CO (l/min), temperature (°C), urine output (ml), arterial pH, lactate (mmol/l), Pco_2_ in arterial blood (mmHg), partial pressure of O_2_ in arterial blood (Po_2_; mmHg), base excess (mEq/l), hs-cTnT (ng/ml), oxygen saturation (%), hematocrit (%), and LV ejection fraction (LVEF; %).

### Hemodynamic Analyses

#### Signal processing.

ABP, electrocardiogram (ECG), LV pressure (LVP), and right atrial pressure (RAP) were continuously recorded during the experiment. At each time point stationary segments of 7-min length on average were selected. R-R intervals (RRI) and HR time series were extracted from the ECG waveform by means of the ECG Analysis Module available for LabChart software; time series of systolic (SAP), diastolic (DAP) and mean (MAP) arterial pressure were obtained from the ABP waveform with specific algorithms (68, 74). Beat-to-beat RAP time series consist of the mean values of RAP within each cardiac cycle; the maximum of the derivative of LVP upstroke (dP/d*t*_max_) was derived on a beat-to-beat basis from the LVP recording, and it was taken as an indirect measure of heart contractility. Temporal relationships were maintained among the time series: given R(*i*) as the R peak of the current beat, RRI(*i*) designated the difference between R(*i* + 1) and R(*i*), SAP(*i*) follows R(*i*) and is followed by DAP(*i*), dP/d*t*(*i*) is the slope of the upstroke right after R(*i*), and RAP(*i*) is the averaged RAP values within RRI(*i*). Finally, an adaptive filter was applied to the data to remove outliers and irregularities (71). Each segment was subdivided into 3-min 50% overlapping windows. Each time series was resampled at 2 Hz by means of zero-order hold techniques, and then it was detrended with a high-order polynomial function to guarantee stationarity, further verified with the Dickey-Fuller and Kwiatkowski-Phillips-Schmidt-Shin statistical tests.

All the indexes obtained for these series were averaged and considered for successive statistical comparisons.

#### Time and frequency indexes.

We computed the mean value of SAP, DAP, MAP, RRI, dP/d*t*_max_, and RAP series.

Spectral indexes obtained from power spectra included low-frequency (LF, 0.04–0.15 Hz) power distribution, total power (TP), which represents the total area under the spectrum and is a measure of the overall variability of the series, and LF%, which represents the relative power in the LF band and is computed as LF/(TP − VLF)%, where VLF is the very low-frequency component (0–0.04 Hz).

#### Cardiac baroreflex sensitivity analysis.

We adopted the bivariate model method (4). Briefly, the two parameters of interest are the feedback (FB) gain, which represents the baroreflex mediated by the ANS, i.e., changes in RRI induced by oscillations in SAP and mediated by the ANS, and the feedforward (FF) gain, which denotes the mechanical influence of RRI on SAP through the heart and vasculature, also called the runoff effect. As the current RRI(*i*) cannot immediately affect SAP(*i*), a one-beat delay was considered for the FF relationship. Granger causality from SAP to RRI and vice versa was verified before computation of the gains (32, 54). The order of the model was optimized based on the Akaike information criterion, ranging from 5 to 15.

#### Heart rate complexity analysis.

We assessed HRV by means of linear and nonlinear methods. In particular, we computed the root mean square of successive differences between adjacent RRI (RMSSD), the standard deviation of successive differences between adjacent RRI (SDSD), and the standard deviation of the overall RRI time series (SD) (48a). Moreover, we calculated the quadratic sample entropy (QSE) to investigate the nonlinear characteristics of HRV (43). The template length *m* was fixed at 1, and the tolerance *r* was computed as 20% of the SD of the time series.

#### Model-based system identification of DAP variability.

To better investigate the different mechanisms of peripheral resistance control, we implemented a multi-input single-output causal black-box model for the prediction and spectral decomposition of DAP beat-to-beat fluctuations from variability signals of SAP, RAP, and RRI. The methodological principles underlying multivariate linear black-box modeling have been exhaustively illustrated elsewhere (5). Assuming that DAP variability is a surrogate measure of peripheral vascular resistance (2, 51), the current DAP value is assumed to be influenced by arterial and cardiopulmonary baroreflex control and by the mechanical coupling between the heart and the circulatory system, the so-called runoff effect. To disentangle these mechanisms and to highlight their different contributions in DAP variability, we modeled such mechanisms as follows:

(1) $$\begin{array}{c} \begin{array}{c} \begin{array}{c} \text{DAP}(i)={\overset{n}{\underset{j=s1}{\sum }}}_{}^{}h_{\text{sap}}\left(j\right)\cdot \text{SAP}\left(i-j\right)+{\overset{n}{\underset{j=s2}{\sum }}}_{}^{}h_{\text{rap}}\left(j\right)\cdot \text{RAP}\left(i-j\right)+h_{\text{rr}}\left(1\right)\cdot \text{RR}\left(i\right)+e\left(i\right) \end{array} \end{array}\\ \;={\text{DAP}}_{\text{sap}}+{\text{DAP}}_{\text{rap}}+{\text{DAP}}_{\text{rr}}+{\text{DAP}}_{\text{noise}} \end{array}$$

We limited the effect of the mechanical runoff to a single gain parameter *h*_rr_(1), for physiological reasons. Granger causality among the cardiovascular time series was verified before computation of the model (32). The optimal model order *n* was assessed with the “ARMA Parameter Reduction algorithm,” as proposed in Ref. 53, starting from a maximal order value *n* equal to 12 and delays [*s*1, *s*2] equal to zero. Model parameters were determined by a least-squares minimization procedure. The black-box input-output relationships in *Eq. 1* can be assumed to be representative of the following mechanisms of DAP variability control:

•  SAP → DAP (DAP_sap_) represents the black-box model for the arterial baroreflex mediated sympathetic control of vasomotor tone.
•  RAP → DAP (DAP_rap_) represents the black-box model of vasomotor tone control related to the cardiopulmonary baroreflex; we assumed that RAP oscillations are representative of pressure oscillations sensed by cardiopulmonary baroreceptors.
•  RRI → DAP (DAP_rr_) represents the mechanical effect of diastolic runoff.

The residual error (DAP_noise_) includes all the remaining sources of variability not measured, such as the influence of DAP past values, the autoregulation-mediated control of peripheral resistance, possible errors, or noise.

To quantify the amount of DAP variability explained by each component, a spectral decomposition was performed. In particular, we performed spectral analysis of the model component series (DAP_sap_, DAP_rap_, DAP_rr_) and then assessed the ratio of LF power of DAP_sap_, DAP_rap_, or DAP_rr_ components over total LF power DAP (LF DAP_sap_/LF DAP, LF DAP_rap_/LF DAP, or LF DAP_rr_/LF DAP), respectively.

#### Analysis of response to phenylephrine and epinephrine administration.

Changes in HR with respect to changes in MAP were analyzed during phenylephrine injection to quantify the ANS-mediated response to the stimulus (37).

Phenylephrine acts directly at the vessel level as a selective α1-adrenergic receptor agonist. In physiological conditions, a bolus of phenylephrine is expected to cause a rise in MAP accompanied by a decrease in HR mediated by the baroreflex autonomic mechanism.

Changes in dP/d*t*_max_ during the injection of epinephrine were analyzed to assess the ANS-mediated response of heart contractility.

### Metabolomics Analyses

Metabolic profile was obtained from tissue samples of the LV for both shock- and sham-treated animals. Sample tissue preparation was performed as reported previously (10). Briefly, frozen tissue samples were disintegrated with a Mikro-Dismembrator S at 3,000 revolutions/min for 40 s. The powder obtained was resuspended in ice-cold MeOH (3 µl/mg tissue) and homogenized for 1 min. Homogenized samples were subsequently centrifuged for 15 min at 10,000 *g*, and supernatants were stored at −80°C. Thirty microliters of each supernatant was used for targeted metabolomics analysis. A targeted quantitative approach using a combined direct flow injection and liquid chromatography (LC) tandem mass spectrometry (MS/MS) assay (AbsoluteIDQ 180 kit; Biocrates, Innsbruck, Austria) was applied to metabolomics analysis. The assay quantifies 188 metabolites from five analyte groups: acylcarnitines, amino acids, biogenic amines, hexoses (sum of hexoses), phosphatidylcholines, and sphingomyelins. The method combines derivatization and extraction of analytes with the selective MS detection using multiple reaction monitoring pairs. Samples were analyzed with an LC/MS (Triple quad 5500; AB Sciex) method (for analysis of amino acids and biogenic amines) followed by flow injection analysis-MS (analysis of lipids, acylcarnitines, and hexose). Methodological details and data preprocessing have been extensively reported in our previous articles (13, 29).

### Statistical Analyses

We adopted the Mann-Whitney *U*-test, also known as the Wilcoxon rank sum test, to verify significant differences in the index values between the two groups (shock and sham-treated) separately at each time point, whereas we used the Friedman test to assess significant changes among time points within the same group of animals. Significance was considered at *P* < 0.05.

## RESULTS

### Hemodynamic Analysis and ANS Indexes

**Fig. 1.**
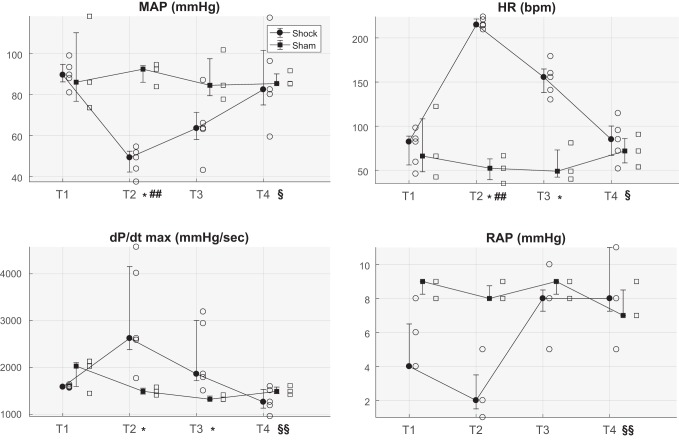
Distributions (median, 25th and 75th percentiles) of the averaged value at each time point of the following variables: mean arterial pressure (MAP), heart rate (HR), maximum of 1st derivative of left ventricular pressure over time (dP/d*t*_max_), and right atrial pressure (RAP) for both populations of hemorrhagic shock- and sham-treated animals. Open symbols indicate values relating to each shock- or sham-treated pig. T1, baseline; T2, after development of shock; T3, after fluid and vasopressor resuscitation; T4, after blood reinfusion; bpm, beats per minute. *P < 0.05 shock- vs. sham-treated animals (Mann-Whitney *U*-test); ^##^*P* < 0.01 vs. T1; §*P* < 0.05, §§*P* < 0.01 vs. T2 (Friedman test, only for shock-treated pigs).

Bleeding caused a significant decrease in MAP, CO, and filling pressures and a concomitant significant increase in HR, heart contractility (dP/d*t*_max_) and lactate compared with sham-treated animals (Fig. 1, Table 1). After resuscitation with fluids and norepinephrine, MAP recovered to the target of 60 mmHg in all animals except one, but HR and heart contractility remained significantly higher than baseline values. Only after resuscitation with shed blood did all the indexes return to baseline values (Fig. 1).

**Table 1.** Clinical and laboratory data for hemorrhagic shock- and sham-treated animals at each time point

	T1	T2	T3	T4
Hct, %				
Shock	23 (22.75, 29.5)	21 (20, 23.75)	10 (10, 11)**^##^**	20 (17.75, 25.5)
Sham	26 (25.3, 27.5)	25 (23.5, 28)	26 (23.8, 26)*	25 (24.3, 25)
CO, l/min				
Shock	3.6 (2.4, 3.8)	1.5 (1.5, 1.8)	5 (4, 5.5)**§§**	2.6 (2.1, 3.9)
Sham	3.5 (3, 4.5)	3.3 (3.2, 3.4)*	3.2 (3.1, 3.6)	3.6 (3.5, 3.8)
LVEF, %				
Shock	59.9 (57.8, 66.6)	72.5 (68.3, 76.9)	64 (62.1, 68.9)	62 (52.5, 69.4)
Sham	60.5 (56.5, 60.9)	60.9 (60.2, 66.9)	64.4 (57.8, 68.2)	57.1 (54.2, 61.4)
Lactate, mmol/l				
Shock	1.8 (1.5, 2.2)	8.5 (7.9, 8.8)**^#^**	5.9 (5.1, 5.9) (*n* = 4)	2.1 (1.6, 2.3)**§**
Sham	1.2 (0.9, 2.5)	0.9 (0.8, 1.7)*	0.8 (0.6, 1.3)	0.8 (0.7, 1.1)*
hs-cTnT, ng/l				
Shock	13.9 (9.4, 33.1)	145.4 (124.3, 589.6)**^##^**	105.2 (86.6, 508.5)**^#^**	61.9 (28.9, 89.8)
Sham	3 (3, 12.5)	4.9 (3.5, 14)*	3.9 (3.2, 40.4)*	14.7 (9.6, 15.1)*
Urine output, ml				
Shock	130 (107.5, 205) (*n* = 3)	7.5 (0, 20) (*n* = 4)	260 (170, 285) (*n* = 4)	220 (125, 342)
Sham	180 (97.5, 270)	60 (85.5, 97.5)	70 (47.5, 85)	60 (60, 75)
Temperature, °C				
Shock	37.9 (37.6, 38.2)	38.3 (38, 39)	36.5 (35.8, 36.9)	36.1 (35.5, 36.7)**§§**
Sham	37.5 (36.8, 37.7)	37 (36.3, 37.4)	37.2 (36.3, 37.4)	37.3 (36.3, 37.5)
pH				
Shock	7.5 (7.49, 7.51)	7.35 (7.3, 7.39)	7.35 (7.23, 7.37)**^##^**	7.34 (7.33, 7.43)
Sham	7.5 (7.45, 7.5)	7.5 (7.49, 7.5)*	7.51 (7.47, 7.51)*	7.47 (7.43, 7.52) (*n* = 2)
Pco_2_, mmHg				
Shock	36.2 (34.4,39.4)	39.7 (35.8,40.6)	39.3 (38.1,46.5)	47.2 (41.6,48.7)**^#^**
Sham	40.6 (37.5, 40.9)	39.6 (39.6, 42,2)	41.4 (39.6, 41.7)	41.7 (37.8, 45.6) (*n* = 2)
Po_2_, mmHg				
Shock	97 (88.8, 126.3)	146 (142.8, 157.5)	164 (148.5, 166)**^#^**	131 (109.8, 155.5)
Sham	89 (80.8, 95)	146 (119, 153.5)	133 (112.8, 151)	124.5 (110, 139)

Values are medians (25th, 75th percentiles). Hct, hematocrit; CO, cardiac output; LVEF, left ventricular ejection fraction; hs-cTnT, plasma high-sensitivity cardiac troponin T; Pco_2_, partial CO_2_ pressure; Po_2_, partial O_2_ pressure; T1, baseline; T2, after development of shock; T3, after fluid and vasopressor resuscitation; T4, after blood reinfusion. Comparisons between shock and sham:

* *P* < 0.05 (Mann-Whitney *U*-test). Comparisons between time points:

# *P* < 0.05,

## *P* < 0.01 vs. T1;

§ *P* < 0.05,

§§ *P* < 0.01 vs. T2 (Friedman test).

The high level of lactate in HS pigs confirmed the severity of the hypovolemic condition. However, the preserved ejection fraction (LVEF) suggested that the severity of shock was not sufficient to induce a concurrent acute heart failure.

Table 2 shows that LF power of BP components (SAP, DAP, MAP) decreased during shock (T2) and remained lower with respect to baseline after fluid and vasopressor resuscitation (T3). After blood reinfusion (T4) the values returned similar to baseline and higher than T3. A similar trend was observed for LF power of HR and dP/d*t*_max_, even if less marked. Baroreflex FB gain was reduced at T2 and T3 (significantly at T2) and recovered at T4; the opposite trend was observed for FF gain.

**Table 2.** Frequency indexes and baroreflex gains for hemorrhagic shock pigs evaluated at each time point

	T1	T2	T3	T4
SAP				
LF power, a.u.	105.3 (39.9, 165.3)	18 (12.2, 29.6)	12.5 (6.3, 35.8)**^#^**	78.9 (48.8, 102.1)
LF % power, %	22.2 (7.2, 39.4)	4.8 (2.4, 6.2)	4.1 (3.3, 12.8)	18 (14.5, 21.7)
DAP				
LF power, a.u.	155.2 (93, 234.7)	37.9 (24.7, 46.7)**^#^**	39.9 (33.5, 71.3)	196.3 (110.6, 248.3)**§**
LF % power, %	38.1 (20.5, 48.4)	11.4 (5.6, 11.7)	11.6 (7.1, 20.8)	53.6 (32.6, 60.7)**§**
MAP				
LF power, a.u.	80.3 (63.7, 123.5)	25.2 (18.1, 41.2)	24.8 (9.6, 47.6)	119.1 (99.1, 174.8)**§****†**
LF % power, %	16.6 (13.5, 29.8)	8.7 (4.2, 10.4)	7.4 (5.9, 13.1)	29.3 (26.4, 43.3)**§**
RRI				
LF power, a.u.	171.5 (161.7, 233)	97.3 (73.2, 130.5)	125.8 (75, 142)	116.3 (104.6, 172.7)
LF % power, %	42.8 (33.4, 57.6)	32.2 (24.9, 42.6)	31.1 (21.6, 36.6)	30 (25.2, 41.2)
dP/d*t*_max_				
LF power, a.u.	147.8 (123.7, 214.6)	75 (50.1, 118.7)	104 (45.6, 133.8)	152.1 (111.3, 202.1)
LF % power, %	31.6 (25.8, 44.9)	21.3 (14.1, 29.7)	22.7 (7.1, 40.9)	30.1 (22.6, 56.7)
BRS				
FB gain, ms/mmHg	3.2 (1.1, 11.4)	0.08 (0.06, 0.2)**^##^**	0.2 (0.1, 0.6)	1.1 (0.8, 5.7)**§**
FF gain, mmHg/ms	0.05 (0.03, 0.1)	0.3 (0.2, 1.6)	0.4 (0.2, 0.5)	0.03 (0.02, 0.1)**§**

Values are medians (25th, 75th percentiles). SAP, systolic arterial pressure; DAP, diastolic arterial pressure; MAP, mean arterial pressure; RRI, R-R interval; BRS, baroreflex sensitivity; FB, feedback; FF, feedforward; T1, baseline; T2, after development of shock; T3, after fluid and vasopressor resuscitation; T4, after blood reinfusion. Comparisons between time points:

# *P* < 0.05,

## *P* < 0.01 vs. T1;

§ *P* < 0.05 vs. T2;

† *P* < 0.05 vs. T3 (Friedman test).

Table 3 reports the HRV indexes. Variability of RRI was dramatically reduced at T2 and did not recover at T3, after fluid and vasopressor administration.

**Table 3.** HRV and HR complexity indexes for hemorrhagic shock pigs evaluated at each time point

	T1	T2	T3	T4
RMSSD, ms	62.4 (37.8, 302.1)	9 (6.4, 16.6)**^#^**	20.7 (16.8, 22.5)	58.3 (34.5, 145.1)**§**
SDSD, ms	3.3 (2, 15.8)	0.5 (0.3, 0.8)**^#^**	1.1 (0.9, 1.2)	2.3 (1.8, 7.6)**§**
SD, ms	17.9 (5.4, 34.4)	1.4 (1.1, 2.7)**^#^**	2.8 (2.6, 4.5)	14.1 (6.2, 15.6)**§**
QSE	2.4 (1.9, 3.5)	−0.1 (−0.4, 0.8)**^#^**	0.9 (0.4, 1.1)	2.3 (1.8, 3.1)**§**

Values are medians (25th, 75th percentiles). HR, heart rate; HRV, HR variability; RMSSD, root mean square of successive difference; SD, standard deviation; SDSD, standard deviation of successive difference; QSE, quadratic sample entropy; T1, baseline; T2, after development of shock; T3, after fluid and vasopressor resuscitation; T4, after blood reinfusion. Comparisons between time points:

# *P* < 0.05 vs. T1;

§ *P* < 0.05 vs. T2 (Friedman test).

The ANS response to the administration of phenylephrine in shock pigs was quantified by considering the amplitude of the change in MAP and HR during the administration of the bolus. Similarly, the response to epinephrine was analyzed by considering the magnitude of the increase in dP/d*t*_max_ during bolus injection. Both MAP and HR variations in response to phenylephrine and dP/d*t*_max_ variation in response to epinephrine were greatly reduced with respect to baseline at the end of the shock period (Table 4).

**Table 4.** Delta values of MAP, HR, and dP/dt_max_ during administration of phenylephrine or epinephrine for shock animals at each time point

	T1	T2	T3	T4
*Responses to phenylephrine administration*				
ΔMAP, mmHg	33.5 (27.7, 41.7)	10.6 (6.1, 13)**^#^**	27.5 (16, 31.5)	27 (19.4, 29.5)
ΔHR, beats/min	−13.4 (−27, −9.1)	0.08 (−5.4, 1.9)**^#^**	−5.1 (−10.5, −0.6)	−10.1 (−12.5, −8.2)
*Responses to epinephrine administration*				
ΔdP/d*t*_max_, mmHg/s	3,401.2 (2,678.2, 3,552.7)	1,602.7 (946.1, 1,986.5)^#^	2,188.6 (1,983.2, 3,082.3)	2,349.2 (1,826.8, 2,515.6)

Delta values (i.e., difference between final value and starting value) are medians (25th, 75th percentiles). MAP, mean arterial pressure; HR, heart rate; dP/d*t*_max_, maximum 1st derivative of left ventricular pressure over time; T1, baseline; T2, after development of shock; T3, after fluid and vasopressor resuscitation; T4, after blood reinfusion. Comparisons between time points:

# *P* < 0.05 vs. T1.

**Fig. 2.**
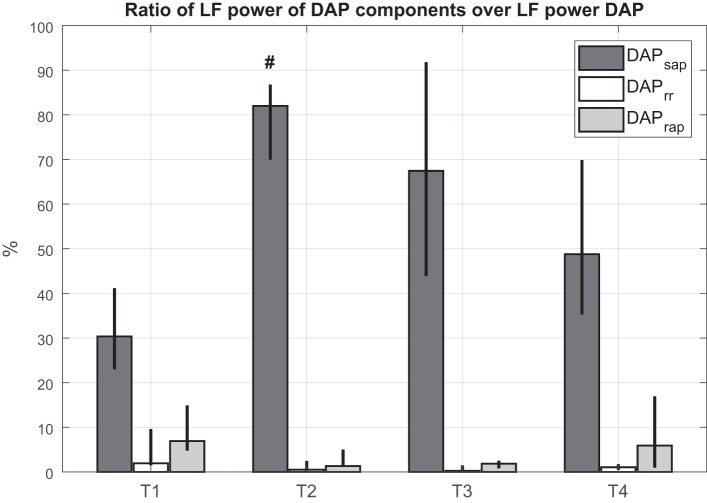
Ratio between low-frequency (LF) absolute power of each predicted component and LF absolute power of diastolic arterial pressure (DAP) at each time point for hemorrhagic shock animals. Column height is median value for the population; black bars indicate values of 25th and 75th percentiles. T1, baseline; T2, after development of shock; T3, after fluid and vasopressor resuscitation; T4, after blood reinfusion; SAP, systolic arterial pressure; RR, R-R interval. ^#^*P* < 0.05 vs. T1 (Friedman test).

As regards the DAP model, the portion of DAP variability mediated by the arterial baroreflex mechanism (LF DAP_sap_/LF DAP) was predominant with respect to the other mechanisms in each phase of the experiment (Fig. 2). A significant increase in LF DAP_sap_/LF DAP was observed in shock with respect to baseline. Furthermore, the mechanical influence of the heart on the circulatory system (LF DAP_rr_/LF DAP) and the portion of DAP variability regulated by the cardiopulmonary baroreflex (LF DAP_rap_/LF DAP) presented a u-shaped trend: they decreased during the shock period, remained lower than baseline after resuscitation, and recovered to baseline values after blood reinfusion.

### Left Ventricle Metabolic Profiling by Targeted Metabolomics

All the measured metabolites are reported in Supplemental Table S1 (Supplemental Material for this article is available online at the Journal website). Only 11 metabolites significantly changed concentrations between sham-treated and HS pigs in LV tissue (Table 5). LV tissue of HS animals was mainly characterized by enhanced levels of long-chain phosphatidylcholine (PC) species and reduced concentrations of a few amino acids compared with LV tissue from sham-treated animals.

**Table 5.** Concentration values of metabolites significantly different in LV tissue between sham- and hemorrhagic shock-treated animals

	Shock	Sham
PC aa C34:4	0.7 (0.4, 0.8)	0.3 (0.2, 0.3)
PC aa C36:4	11.9 (7.8, 17.9)	4.8 (3.2, 5.2)
PC aa C36:5	2.6 (1.7, 3.4)	0.9 (0.7, 1.1)
PC aa C38:4	1.8 (1.2, 2.3)	0.7 (0.4, 0.8)
PC aa C38:6	1.8 (1.2, 2.3)	0.65 (0.57, 0.66)
PC ae C38:0	0.3 (0.2, 0.4)	0.13 (0.1, 0.13)
PC ae C42:3	0.08 (0.04, 0.09)	0.029 (0.028, 0.03)
Arginine	11.7 (6.8, 14.3)	26.3 (24.4, 28.2)
Isoleucine	6.5 (5.9, 9.1)	11.9 (11.7, 13.8)
Ornithine	2.5 (1.8, 3.04)	4.4 (4.2, 5.3)
SM C22:3	0.1 (0.09, 0.14)	0.07 (0.05, 0.07)

Values (in μm) are medians (25th, 75th percentiles). LV, left ventricular; PC aa Cxx:x, phosphatidylcholine diacyl C xx:x; SM C22:3, sphingomyelin C 22:3.

## DISCUSSION

### Cardiovascular Indexes and Models

The trends of vital signs and lactate confirmed the hypovolemic shock associated with a hyperdynamic cardiovascular response (Fig. 1, Table 1).

Changes in LF oscillations of BP can be related to changes in the outflow of the sympathetic nervous system, and spectral analysis of BP has been proven to be a powerful tool for identification of the different cardiovascular control mechanisms that regulate BP (65, 66). From this perspective, the reduction of LF power we observed during shock (Table 2) can be interpreted as a withdrawal or a saturation of sympathetic activity (6, 16, 25, 41, 64). Convertino et al. (22) and Cooke et al. (25) measured muscle sympathetic nerve activity during increasing negative pressure in a lower body negative pressure protocol, supporting the hypothesis that sympathetic withdrawal may represent a fundamental mechanism for the development of circulatory shock. Our results are thus in line with this hypothesis, i.e., acute bleeding and HS may cause a cardiovascular collapse characterized by a depressed peripheral sympathetic outflow.

Many hypotheses have been formulated in the past years, trying to explain this phenomenon. Some literature supports the concept that an “empty heart” might contribute to cardiac receptor stimulation, resulting in the activation of cardiac vagal afferents and subsequent sympathetic depression (50, 62, 64). Koyama et al. (41) proposed that prolonged brain ischemia could be the triggering cause of sympathetic outflow depression documented in hypovolemic shock. Another possible mechanism that may contribute to the loss of sympathetic peripheral outflow is the resetting of baroreflexes, which leads to a loss of synchrony between arterial BP and muscle sympathetic nerve activity, suggesting an impairment of arterial baroreflex control over sympathetic vasomotor activity (20, 24, 39, 40).

The available data and the results of our study do not fully support one of the above mechanisms. However, the hypothesis of prolonged brain ischemia-mediated sympathetic depression can be excluded since the shock condition was not prolonged for long and all animals recovered after blood reinfusion. Interestingly, after resuscitation with fluid and vasopressor (T3) the level of LF power did not recover to baseline value despite the animals reaching the target MAP values, and CO was higher than baseline (Table 1). Therefore, the persisting depressed LF oscillations of BP cannot be attributed to vagal reflexes activated by an unloading of cardiopulmonary baroreceptors.

As regards the cardiac baroreflex sensitivity (BRS) we observed a significant decrease of sensitivity during shock (BRS FB gain) that persisted even after the resuscitation maneuvers at T3 (Table 2). A dynamic reduction in BRS with decreased central blood volume has already been reported (19, 21, 23, 28, 47, 57, 73), and since it is known to reflect baroreflex-mediated cardiac vagal withdrawal (23) it could be explained as a compensatory mechanism that leads to a greater tachycardia reserve (18).

Interestingly, the opposite trend was observed for the feedforward gain (BRS FF gain), or runoff effect, which was higher compared with baseline both in shock (T2) and after fluid and vasopressor resuscitation (T3). This may be due to a markedly high HR.

The black-box model of DAP variability, taken as a surrogate of peripheral vascular resistance, highlighted interesting changes in peripheral control mechanisms induced by severe blood loss. During shock the cardiac baroreflex-mediated DAP oscillations (DAP_sap_) significantly increased with respect to baseline, passing from ~30% to 80% in all animals. The other components (DAP_rap_, DAP_rr_) decreased at T2 and T3 with respect to baseline (Fig. 2). These results highlighted that the large reduction in peripheral vascular resistance can be explained again by the high HR, which cannot sustain BP changes.

The results of phenylephrine and epinephrine maneuvers confirmed a reduced sympathetic modulation of HR and BP as previously reported. During shock there was a reduced responsiveness to the drugs, mostly in the HR: indeed, the variation during phenylephrine administration was negligible (Table 4).

Analysis of HRV revealed that HS induced a decrease in HRV and HR complexity. In this context, the notion of complexity, as calculated by means of entropy estimates, refers to the regularity/irregularity of the time series, i.e., the degree to which template patterns repeat themselves; repeated patterns imply regularity and lead to reduced values of entropy. A loss of HR complexity, which means a higher regularity of the HR, is considered a feature of impaired adaptation of the ANS to physiological stress in several cardiovascular pathologies (42, 46, 48). Accordingly, our results show that the autonomic modulation of HR in response to acute stress induced by hemorrhage and shock was severely impaired (Table 3), as already reported in other studies (7). Interestingly, this condition is not relieved after administration of fluids and vasopressors but only after reinfusion of shed blood.

HRV is assumed to be mostly dependent on the parasympathetic outflow to the heart; moreover, the recovery of baroreflex functionality, which followed the same trend as HRV during the experiment, has also been associated with an increased vagal activity in several pathologies (44). Thus we could suggest a vagal driver of effective resuscitation (61). Furthermore, an extensive literature has recently pointed to the central role of the vagus nerve as therapeutic target: direct vagus nerve stimulation has been proven to be beneficial in several diseases, including hemorrhage and shock (8, 26).

The lack of a recovery in the ANS control after resuscitation with fluid and vasopressor could be explained by the fact that the massive fluid infusion produced a reduction in the arterial blood oxygen content and, consequently, in the delivery of oxygen to peripheral tissues. Indeed, the hematocrit was significantly reduced and blood lactate still high at T3 (Table 1), supporting the notion of an ongoing mismatch between whole body oxygen consumption and oxygen delivery. Furthermore, the time window between T2 and T3 may be too short to obtain a complete resolution of the oxygen debt. Finally, the volume expansion offered by fluid infusion is limited because of redistribution of fluids in the interstitium and excretion in urine that usually occur soon after the beginning of fluid resuscitation (36).

After the transfusion of the shed blood we observed a complete restoration of HR and BP variability, lactate clearance, and hemodynamics to near-baseline values. Probably the increase in hematocrit and arterial oxygen content was effective in restoring tissue oxygenation. Another hypothesis, based on the study of hemodilution physiology, is that the restoration of systemic and microvascular conditions after hemorrhage followed by hypovolemic shock depends mostly on blood rheological properties rather than on maintenance of oxygen-carrying capacity. The rationale for this hypothesis originates from experimental studies in HS, showing that a threshold of blood/plasma viscosity is required to maintain microvascular perfusion and, in particular, functional capillary density (11).

### Cardiac Functionality and LV Tissue

The shock protocol produced a clear decrease in CO and MAP and a hypoxic condition as supported by a lactate increase above 8 mmol/l. LVEF, however, was preserved, with an increased cardiac contractility during shock. After fluid resuscitation (T3) LVEF returned to baseline value, but only after blood reinfusion (T4) did both CO and dP/d*t*_max_ recover to values similar to baseline.

These results suggest that the shock insult did not impair cardiac contractility. The main reason for this result could be the short period of shock before initiation of treatments. Indeed, in our study shock was maintained up to 2 h after confirmation of lactate increase, while in previous experimental studies of HS the onset of myocardial depression was usually observed between 2 and 5 h after insult (49, 55). In our study, shock was not sufficiently prolonged to significantly impair cardiac contractility and to induce acute heart failure.

Nevertheless, plasma hs-cTnT increased significantly in shocked animals, remained high after fluid resuscitation, and finally returned to baseline values after blood reinfusion. The severity and the kinetics of troponin release do not support the hypothesis of an acute coronary syndrome (1, 31); indeed, the peak plasma concentration observed in shock was only modestly increased. Probably such an increase in hs-cTnT is the result of a general ischemic sufferance of cardiomyocytes. The most plausible hypothesis may consist in the mismatch between oxygen delivery and oxygen consumption, leading to a type II myocardial ischemia. In fact, during hemorrhage and resuscitation with fluids, several factors may determine decrease in myocardial oxygen delivery (hypotension, low CO, reduced diastolic time, reduced hematocrit following fluid resuscitation) and increase in myocardial oxygen consumption (extreme tachycardia, circulating catecholamines, both endogenous and administered as support, i.e., norepinephrine) (70).

Another possible explanation of the increase in hs-cTnT could be the mechanical stress on the LV wall in the condition of elevated myocardial contractility and low filling volume, which causes sarcomere disruption in a highly susceptible spot (i.e., “zonal lesion”) (35). Unfortunately, we did not investigate the presence of ischemic lesions in histological samples of the LV, so we cannot confirm this hypothesis.

As regards metabolomics findings, only a small subset of the measured metabolites were differently abundant in LV tissue between HS and sham treatment (Table 5). A disturbance in the composition of myocardial membrane PC species occurred. Indeed, shocked LV tissue presented a marked increase in PC species, containing long-chain polyunsaturated fatty acids, such as PCaaC34:4, PCaaC36:4, and PCaaC36:5, with further elongation/desaturation products. We can speculate that since long-chain polyunsaturated fatty acids reduce T cell activation and dampen inflammation (12), their enhanced presence would help relieve serious inflammatory conditions.

Concomitantly, raised plasmalogen levels such as PCasC38:0 and PCec42:3, which act as endogenous antioxidants (9), may protect cardiomyocytes during hypoxia in shocked LV. Interestingly, plasmalogens, highly predominant in the sarcolemma and sarcoplasmic reticulum of myocardial cells (33), play an important role in regulating myocardial electrical excitability (72) and sodium/calcium exchange in cardiac sarcolemmal preparations (30).

Together these findings converge toward a plausible adaptation of the LV metabolic asset to cope with the insult. Such change in the metabolic asset might also be viewed as a key mechanism for either membrane structural rearrangement or damage to cardiomyocytes (45) and might directly or indirectly contribute to the production of electrophysiological abnormalities and arrhythmogenesis.

Moreover, shocked LV tissue presented a dysregulated arginine metabolism, characterized by reduced arginine and ornithine levels, thus suggesting a possible imbalance of nitric oxide (NO) formation. In heart, alterations of the arginine-NO pathway have been reported in chronic heart failure (63). NO synthesis requires the presence of arginine inside the cells of responsive tissues. Although some cell types can synthesize arginine from ornithine or citrulline, cardiac myocytes cannot produce it, and thus cardiac muscle must import this amino acid from the circulation. Depletion of arginine leads to nitric oxide synthase uncoupling. Combination of $O_{2}^{-}$ with NO from enzymatic or nonenzymatic sources will result in the production of peroxynitrite (ONOO^−^), an oxidizing agent associated with cell damage, decreased myocardial contractility, and congestive heart failure (56).

### Limitations of the Study

The main limitation of this study is the small sample size. Moreover, the short time between T2 (shock), T3 (resuscitation), and T4 (blood reinfusion) could have attenuated the effects of shock in the physiological and metabolic responses. Finally, the unavailability of direct measures of autonomic outflow, such as muscle or cardiac sympathetic nerve activity, did not permit more than speculation about the compensatory mechanisms that are activated or suppressed during shock and resuscitation.

### Conclusions

The results of this study suggest that BP variability, HRV, and baroreflex trends can be valuable tools to evaluate the severity of HS and they may represent a more useful end point for resuscitation, in combination with standard measurements such as mean values and biological measurements. In fact, they can add important insights into the recovery of the autonomic mechanisms of BP and HR regulation, which are fundamental for the recovery of organ dysfunction in shocked subjects. In this study, for example, resuscitation with fluid and vasopressor was effective in restoring circulating volume, CO, and mean BP, but indexes of autonomic functionality revealed that ANS control was not fully recovered. Interestingly, the high concentrations of cardiac troponin and a dysregulated metabolism in the LV tissue after resuscitation are signs of a stress condition induced by shock not resolved by the resuscitation maneuver.

## GRANTS

This study was supported by the European Union FP7 Health Programme, ShockOmics Project, Grant No. 602706.

## DISCLOSURES

No conflicts of interest, financial or otherwise, are declared by the authors.

## AUTHOR CONTRIBUTIONS

G. Baselli and M.F. conceived and designed research; G. Babini and G.R. performed experiments; M.C., R.P., L.B., and M.F. analyzed data; M.C., G. Babini, G.R., R.P., and M.F. interpreted results of experiments; M.C. prepared figures; M.C. drafted manuscript; M.C., G. Babini, G.R., R.P., and M.F. edited and revised manuscript; M.C., G. Babini, G. Baselli, G.R., R.P., L.B., and M.F. approved final version of manuscript.

## Supplemental Data

Supplemental Table Supplemental Table: Dataset of all measured metabolites concentration - .xlsx (237 KB)
